# Supplementary material for: Using network clustering to predict copy number variations associated with health disparities
Source: PeerJ. 2015 Mar 5;3:e677. doi: 10.7717/peerj.677 (PMC4358638; doi:10.7717/peerj.677)
Supplement: Table S1 [file peerj-03-677-s001.docx]

Table S1 Summery of biological networks

| Network | Inflation  Value | Total  Clusters | Cluster Sizes | | | |
| --- | --- | --- | --- | --- | --- | --- |
|  |  |  | Maximum | Minimum | Median | Mean |
| HPRDNet^*^:  9451 genes  36880 interactions | 1.1 | 111 | 9203 | 2 | 2 | 85 |
|  | 1.2 | 128 | 9025 | 1 | 2 | 74 |
|  | 1.3 | 363 | 3454 | 1 | 3 | 26 |
|  | 1.4 | 660 | 432 | 1 | 5 | 14 |
|  | 1.5 | 1036 | 260 | 1 | 4 | 9 |
|  | 1.6 | 1401 | 192 | 1 | 3 | 7 |
|  | 1.7 | 1704 | 152 | 1 | 3 | 6 |
|  | 1.8 | 1990 | 146 | 1 | 3 | 5 |
|  | 1.9 | 2222 | 119 | 1 | 3 | 4 |
|  | 2.0 | 2447 | 90 | 1 | 3 | 4 |
| MultiNet:  14445 genes  109598 interactions | 1.1 | 21 | 14399 | 2 | 2 | 450 |
|  | 1.2 | 22 | 14394 | 2 | 2 | 430 |
|  | 1.3 | 43 | 13614 | 1 | 2 | 220 |
|  | 1.4 | 226 | 5560 | 1 | 3 | 42 |
|  | 1.5 | 600 | 3524 | 1 | 2 | 16 |
|  | 1.6 | 923 | 2402 | 1 | 2 | 10 |
|  | 1.7 | 1387 | 1589 | 1 | 2 | 7 |
|  | 1.8 | 1660 | 1215 | 1 | 2 | 6 |
|  | 1.9 | 1887 | 996 | 1 | 2 | 5 |
|  | 2.0 | 2116 | 804 | 1 | 2 | 4 |

* Self interactions, interactions with unknown genes and ambiguous genes, are not included. The gene number is based on selected interactions.
