# Supplementary material for: Using network clustering to predict copy number variations associated with health disparities
Source: PeerJ. 2015 Mar 5;3:e677. doi: 10.7717/peerj.677 (PMC4358638; doi:10.7717/peerj.677)
Supplement: Table S2 [file peerj-03-677-s002.docx]

Table S2. Results of gene mapping of SNPs and CNV coordinates^1^

|  | **Pathogenic Genes** | **CNV_AA** | **CNV_CA** |
| --- | --- | --- | --- |
| **Total^2^** | 2810 | 194 | 258 |
| **in HPRDNet^3^** | 1791 | 48 | 62 |
| **in MultiNet^3^** | 2143 | 64 | 97 |

1. Since this study focuses on network-derived gene clusters, only genes that are listed in the networks were kept. 2. The number of distinct genes in the query result. 3. The number of genes that are listed in HPRDNet or MultiNet.
