# Supplementary material for: Using network clustering to predict copy number variations associated with health disparities
Source: PeerJ. 2015 Mar 5;3:e677. doi: 10.7717/peerj.677 (PMC4358638; doi:10.7717/peerj.677)
Supplement: Table S3 [file peerj-03-677-s003.docx]

Table S3. Top-ranked clusters from HPRDNet and MultiNet*

| **Network** | **I** | **Cluster No** | **Cluster**  **Name** | **CNV_AA** | **CNV_CA** | **FDR_CNV** | **FDR_OMIM** | **Score** |
| --- | --- | --- | --- | --- | --- | --- | --- | --- |
| **HPRDNet** | 1.1 | **-** |  | **-** | **-** | **-** | **-** | **-** |
|  | 1.2 | **-** |  | **-** | **-** | **-** | **-** | **-** |
|  | 1.3 | **-** |  | **-** | **-** | **-** | **-** | **-** |
|  | 1.4 | 175 | AA1 | *HSPB1* | - | 0.1548 | 3.78×10^-6^ | 5.85×10^-7^ |
|  | 1.5 | 190 | AA1 | *HSPB1* | - | 0.1032 | 1.45×10^-5^ | 1.50×10^-6^ |
|  |  | 429 | CA1 | - | *ATP2A1* | 0.1094 | 1.80×10^-5^ | 1.97×10^-6^ |
|  | 1.6 | 162 | AA2 | *HSPB1* | - | 0.0964 | 4.08×10^-5^ | 3.93×10^-6^ |
|  |  | 470 | CA1 | - | *ATP2A1* | 0.0896 | 0.0369 | 3.31×10^-3^ |
|  | 1.7 | 142 | AA2 | *HSPB1* | - | 0.0842 | 0.0454 | 3.82×10^-3^ |
|  |  | 509 | CA1 | - | *ATP2A1* | 0.0690 | 0.1989 | 1.37×10^-2^ |
|  | 1.8 | 109 | AA3 | *HSPB1* | - | 0.0889 | 0.1010 | 8.98×10^-3^ |
|  |  | 516 | CA1 | - | *ATP2A1* | 0.0756 | 0.1910 | 1.44×10^-2^ |
|  | 1.9 | - |  | - | - | - | - | - |
|  | 2.0 | - |  | - | - | - | - | - |
| **MultiNet** | 1.1 | - |  | - | - | - | - | - |
|  | 1.2 | - |  | - | - | - | - | - |
|  | 1.3 | - |  | - | - | - | - | - |
|  | 1.4 | 85 | CA1 | - | *ATP2A1* | 0.1501 | 0.0289 | 4.34×10^-3^ |
|  | 1.5 | 174 | AA4 | *HSPB1* | - | 0.0979 | 0.0023 | 2.25×10^-4^ |
|  |  | 188 | CA1 | - | *ATP2A1* | 0.1237 | 0.0345 | 4.27×10^-3^ |
|  | 1.6 | 258 | AA4 | *HSPB1* | - | 0.0743 | 0.0035 | 2.60×10^-4^ |
|  |  | 277 | CA1 | - | *ATP2A1* | 0.1708 | 0.0384 | 6.56×10^-3^ |
|  | 1.7 | 320 | AA4 | *HSPB1* | - | 0.0838 | 0.0057 | 4.78×10^-4^ |
|  |  | 351 | CA1 | - | *ATP2A1* | 0.1814 | 0.0467 | 8.47×10^-3^ |
|  | 1.8 | 377 | AA4 | *HSPB1* | - | 0.0809 | 0.0067 | 5.42×10^-4^ |
|  |  | 412 | CA1 | - | *ATP2A1* | 0.1347 | 0.0509 | 6.86×10^-3^ |
|  | 1.9 | 410 | AA4 | *HSPB1* | - | 0.0747 | 0.0078 | 5.83×10^-4^ |
|  |  | 443 | CA1 | - | *ATP2A1* | 0.1159 | 0.0663 | 7.68×10^-3^ |
|  | 2.0 | 479 | CA1 | - | *ATP2A1* | 0.0988 | 0.0728 | 7.19×10^-3^ |

Ranking details of selected clusters were listed. I is the inflation value used in clustering process. Cluster NO is the serial number of a cluster. CNV_AA and CNV_CA are CNV-related genes. The FDR_CNV and FDR_OMIM are FDR values from tests for CNV genes and pathogenic gene enrichment tests, respectively. Score is the multiplication result of FDR_CNV and FDR_OMIM and is used for ranking. *In two cases, CNV_AA and CNV_CA segments cover the same gene and led to ambiguity in the gene’s role in health disparities. We excluded these two genes from our analysis for clarity.
