# Supplementary figures and images for: Using network clustering to predict copy number variations associated with health disparities
Source: PeerJ. 2015 Mar 5;3:e677. doi: 10.7717/peerj.677 (PMC4358638; doi:10.7717/peerj.677)

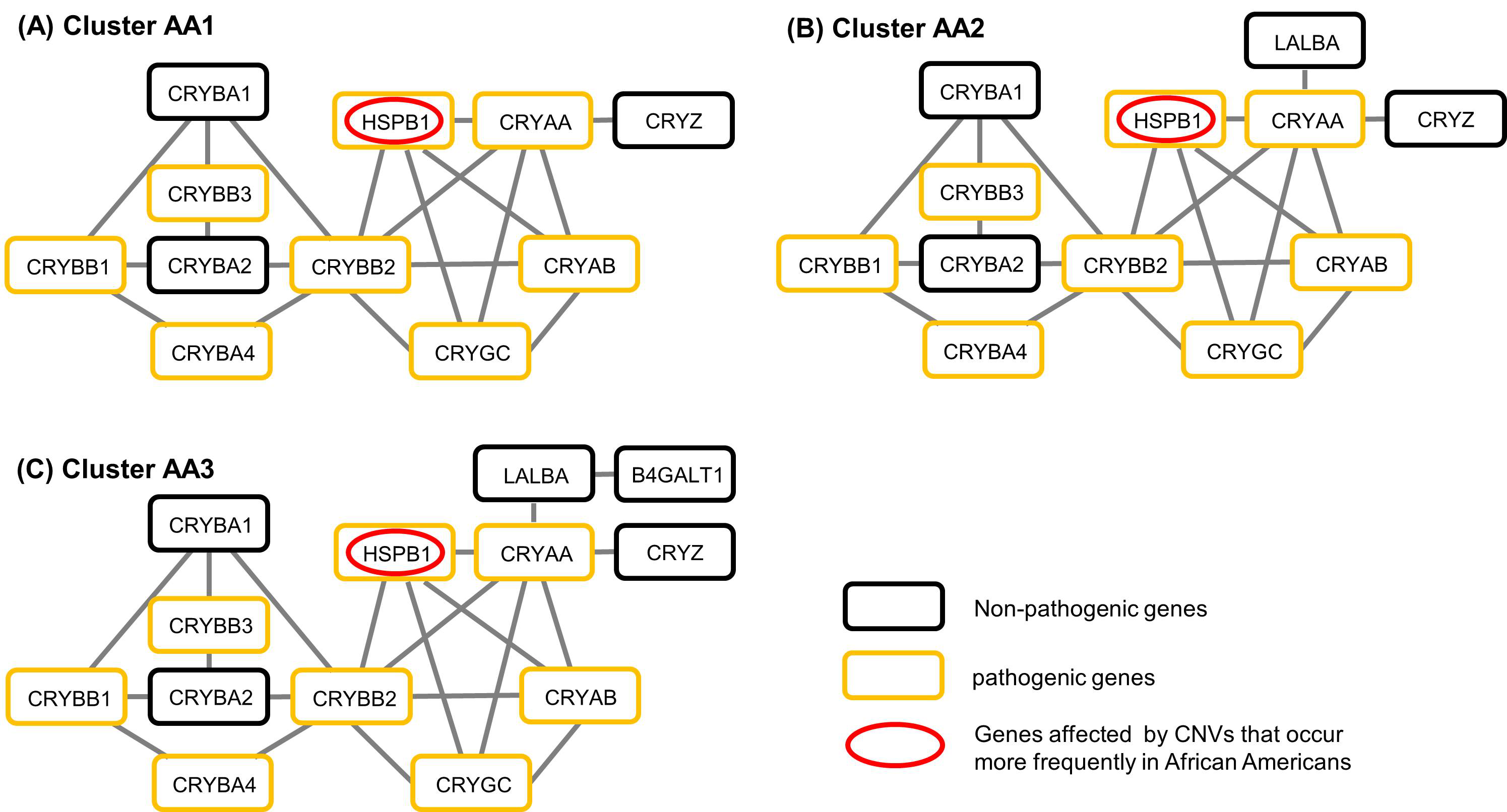

Supplement: Figure S1 — Each rounded rectangle represents a gene and each gray line represents a gene–gene interaction. Black rounded rectangles represent non-pathogenic genes and orange rounded rectangles represent pathogenic genes. Genes labeled with red or blue ovals are located in African American CNVs or in Caucasian CNVs. [file peerj-03-677-s004.png]
